# MiR-137 Targets Estrogen-Related Receptor Alpha and Impairs the Proliferative and Migratory Capacity of Breast Cancer Cells

**DOI:** 10.1371/journal.pone.0039102

**Published:** 2012-06-18

**Authors:** Yuanyin Zhao, Yuping Li, Guiyu Lou, Li Zhao, Zhizhen Xu, Yan Zhang, Fengtian He

**Affiliations:** Department of Biochemistry and Molecular Biology, College of Basic Medical Sciences, Third Military Medical University, Chongqing, China; Institut de Génomique Fonctionnelle de Lyon, France

## Abstract

ERRα is an orphan nuclear receptor emerging as a novel biomarker of breast cancer. Over-expression of ERRα in breast tumor is considered as a prognostic factor of poor clinical outcome. The mechanisms underlying the dysexpression of this nuclear receptor, however, are poorly understood. MicroRNAs (miRNAs) regulate gene expression at the post-transcriptional level and play important roles in tumor initiation and progression. In the present study, we have identified that the expression of ERRα is regulated by miR-137, a potential tumor suppressor microRNA. The bioinformatics search revealed two putative and highly conserved target-sites for miR-137 located within the ERRα 3′UTR at nt 480–486 and nt 596–602 respectively. Luciferase-reporter assay demonstrated that the two predicted target sites were authentically functional. They mediated the repression of reporter gene expression induced by miR-137 in an additive manner. Moreover, ectopic expression of miR-137 down-regulated ERRα expression at both protein level and mRNA level, and the miR-137 induced ERRα-knockdown contributed to the impaired proliferative and migratory capacity of breast cancer cells. Furthermore, transfection with miR-137mimics suppressed at least two downstream target genes of ERRα–CCNE1 and WNT11, which are important effectors of ERRα implicated in tumor proliferation and migration. Taken together, our results establish a role of miR-137 in negatively regulating ERRα expression and breast cancer cell proliferation and migration. They suggest that manipulating the expression level of ERRα by microRNAs has the potential to influence breast cancer progression.

## Introduction

Human breast cancer is a malignant tumor with high level of heterogeneity. Intricate signaling network is the molecular foundation of the malignant progression and heterogeneity formation of breast tumor [1]. Studies in the past decades have revealed several classic signaling molecules involved in breast tumorigenesis, such as estrogen receptor alpha (ERα) [2], progesterone receptor (PR) [3] and human epidermal growth factor receptor-2 (HER2) [4], all of which have been identified as biomarkers for molecular classification of breast cancer and targets of individual therapy of the disease [5], [6], [7].

Nowadays, nuclear receptor estrogen-related receptor α (ERRα) is considered to be another important component of breast cancer signaling network and is emerging as a novel biomarker of the disease [8]. ERRα was originally cloned using the DNA-binding domain (DBD) of ERα as a probe to screen the human cDNA library [9]. Despite its significant homology with ERα, ERRα does not respond to estrogen or estrogen-like molecules. Actually, no endogenous ligand for ERRα has been identified so far. Moreover, crystallographic studies have suggested that the ligand binding domain (LBD) of ERRα can recruit co-regulators in a ligand-independent manner [10], [11]. In another word, ERRα is a constitutively active orphan nuclear receptor. The primary physiological role of ERRα can be viewed as a regulator of energy metabolism, which is required for cell adaption to various stresses and energy needs [12]. Recent studies have been portraying a picture about the implication of ERRα in breast cancer initiation and progression. First, breast cancer tissues express a higher level of ERRα compared to adjacent benign tissues, which is significantly correlated with an increased risk of recurrence and adverse clinical outcome [8], [13], [14]. Second, ERRα interferes with the estrogen signaling pathway both through participating in the local mammary steroidogenesis [15], [16] and through co-regulating a group of genes with ERα [17], [18]. Third, there is a reciprocal relationship between ERRα and HER2 signaling pathway. The transcriptional activity of ERRα can be enhanced by the EGF-HER2 signaling pathway [19], [20]. In turn, activated ERRα can enhance the expression of the HER2 gene ERBB2 [21]. The positive regulatory loop between ERRα and EGF-HER2 pathway is considered to promote the conversion of ERα-positive luminal breast tumor into a more aggressive HER2-positive type [21]. Finally, transcriptome analysis on a genome-wide scale has shown that ERRα can independently regulate the expression of a large number of genes that mediate a range of biological processes, such as metabolism, cell proliferation, cell cycle, apoptosis, metastasis and transcription [17]. By intersecting the ERRα target genes in breast cancer cells with the gene expression profiles of several cohorts of human breast tumors, ERRα signaling is considered to contribute to the heterogeneity of the disease [17].

In summary, ERRα is a signaling molecule widely expressed in different subtypes of breast tumor, which independently and/or coordinately modulates the tumor progression. Therefore, finding an effective approach to manipulate the activity or the expression of ERRα has profound significance for the therapy of breast cancer. Currently, several synthetic compounds have been identified as inverse agonists of ERRα to modulate its transcriptional activity [22], [23], [24], however, the regulatory mechanisms of its gene expression are poorly understood. It was reported that ERRα can regulate the expression of itself through binding to the multiple-hormone response element (MHRE) located within the promoter region of the ERRα gene [25]. The positive auto-regulatory loop is a guarantee for cell to immediately adapt to energy needs for some physiological stresses. Besides ERRα, the ERRγ and ERα were discovered to regulate the ERRα gene transcription through the MHRE [26], [27]. However, besides at transcriptional level, are there any regulatory mechanisms at additional levels? Furthermore, what is the mechanism underlying the up-regulation of the basal level of the ERRα protein in breast tumorigenesis? These issues remain to be elucidated.

MicroRNAs (miRNA) are a class of endogenous, small, non-coding RNAs. Mature miRNAs (generally 18–25 bp nucleotides in length) act as regulators of gene expression at the post-transcriptional level via sequence-specific interaction with the target mRNA [28], [29]. It is estimated that the expression of up to 60% of all protein-coding genes are under the control of miRNAs [30]. Therefore, miRNAs are involved in a range of cellular processes related to carcinogenesis. It has been shown that miRNAs can act as oncogenes or tumor suppressor genes, and aberrant expression of miRNAs occurs in various tumors [31], [32]. In breast cancer, miRNAs signatures are correlated with the biopathologic features of different breast tumor subtypes [33]. To date, numerous miRNAs, such as miR-21, miR-125, miR-200, miR-221/222 and so on, were reported to be aberrantly expressed in breast cancer [34]. The studies aimed at exploring their functions in breast cancer revealed that a lot of signaling molecules including ERα and HER2, were targets of miRNAs [34], [35]. Therefore, uncovering the relationship between miRNAs and key human breast cancer biomarker gene will provide us an additional perspective to recognize the mechanism underlying breast cancer initiation and progression.

The present study is aimed at exploring the potential of regulating the ERRα expression by microRNAs. Our results show that miR-137, a potential tumor suppressor microRNA, can negatively modulate the expression of ERRα and suppress the growth and migration of breast cancer cells partly through two immediate downstream effectors of ERRα-cell cycle protein cyclinE1 and WNT11.

## Results

### The 3′UTR of ERRα mRNA Contains Two Functional Target Sites for miR-137

**Figure 1:**
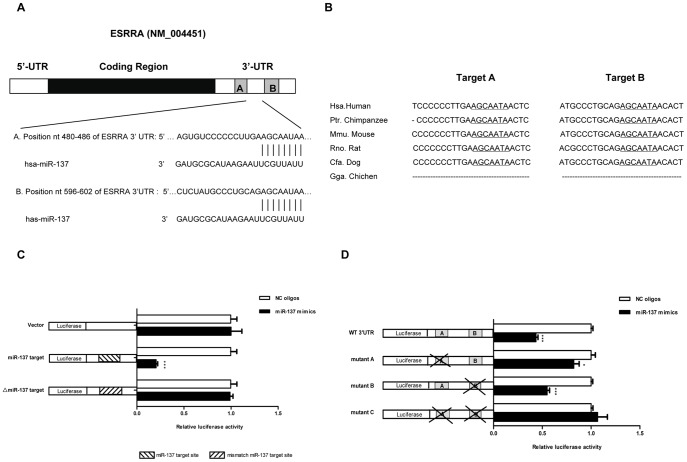
Identification of two highly conserved miR-137 target sites within the ESRRA 3′UTR. A. Schematic representation of the ERRα (ESRRA) mRNA with two putative sites (A and B) targeted by miR-137. B. Sequence alignment of predicted miR-137 target sites located within ESRRA 3′UTR showing high conservation among different species. The sequence of miR-137 target sites in ESRRA 3′UTR is shown in underlined. C. Luciferase reporter assay to verify activity of miR-137 upon the consensus miR-137 target site. HepG2 cells were transfected with Empty reporter plasmids, luciferase constructs containing perfect match miR-137 target site (miR-137 target) or mismatch miR-137 target site (△miR-137 target) and either miR-137 mimcs or NC oligos. Luciferase activity was determined 24 hr after transfection. Relative luciferase expression (firefly normalized to Renilla) values are the ratio of miR-137-treated reporter vector compared with the same NC oligos-treated reporter vector. Data are representative of at least three independent experiments. Error bars: SD. ***: P<0.0001. D. Luciferase reporter assay to evaluate the interaction between miR-137 and 3′-UTR of ESRRA. HepG2 cells were transfected with luciferase constructs containing wild-type (WT 3′UTR) or deletion mutated ESRRA 3′UTR (mutant A, mutant B and mutant C) and either miR-137 mimcs or NC oligos. Luciferase activity was determined 24 hr after transfection. Relative luciferase expression (firefly normalized to Renilla) values are the ratio of miR-137-treated reporter vector compared with the same NC oligos-treated reporter vector. Data are representative of three independent experiments. Error bars: SD. *: p<0.05, ***: P<0.0001.

To identify the miRNAs that target ERRα, we performed a bioinformatics search employing three well-known prediction algorithms (TargetScan [36], PicTar [37] and miRanda [38]). MiR-137 was predicted as a potential microRNA that targets the ERRα gene (ESRRA NM_004451) by these three algorithms. Moreover, two putative target sites (AGCAAUA) for the miR-137 seed sequence (UAUUGCU)were predicted to be located within the ESRRA 3′UTR at nt 480–486 (named target site A ) and nt 596–602 (named target site B) respectively (Fig. 1A). More importantly, both of them are highly conserved across different species (Fig. 1B).

To investigate the interaction between miR-137 and its predicted target sites within ESRRA 3′UTR and to evaluate the relative contribution of each miR-137 binding site to the interaction, we generated a series of dual luciferase reporter plasmids (Fig. 1C and 1D). These included plasmids with perfect miR-137 target sequence (miR-137 target), mismatched miR-137 target site (△miR-137 target), full-length wild-type ESRRA 3′UTR (WT 3′UTR), or mutated ESRRA 3′UTR. At first, we tried to determine whether the synthetic miR-137 mimics could recognize its target site in our reporter assay system. To this end, we employed the reporter plasmid–“miR-137 target” as the systemic positive control and the “△miR-137 target” as the negative control. As shown in Figure 1C, in HepG2 cells (a cell line that expresses relatively low level of endogenous ERRα (Fig. S1)), miR-137 mimics reduced the luciferase activity of plasmid “miR-137 target” by 80%. In contrast, we did not observe that miR-137 reduced the expression of empty plasmid or plasmid with mismatched miR-137 target (△miR-137 target).

We next tested the interaction between miR-137 and the 3′-UTR of ESRRA. Our data showed that compared with NC oligos, miR-137 mimics also dramatically decreased the luciferase activity of reporter plasmid with the intact ESRRA 3′UTR. Furthermore, no matter whether target site A or target site B was deleted (mutant A and mutant B) the decrease of luciferase activity was compromised to a certain extent. As shown in Figure 1D, miR-137 could decrease the luciferase activity of the reporter plasmid with WT 3′UTR to 43% of NC oligos treated group. If site B was deleted, the decreased activity of the reporter plasmid was restored to about 55%, whereas once site A was deleted, the luciferase activity was restored to 78%. Not surprisingly, once both miR-137 target sites were lost (mutant C), the activity of the reporter gene was no longer affected by miR-137 mimics at all. Taken together, these data indicate that ESRRA 3′UTR is a specific direct target of miR-137. The two predicted target sites possess unequal ability to interact with miR-137 (target site A is the major functional miR-137 binding site) but both of them are functional and can mediate the repression of reporter gene expression in an additive manner.

### Breast Cancer Cells Lose miR-137 and Express High Level of ERRα

**Figure 2:**
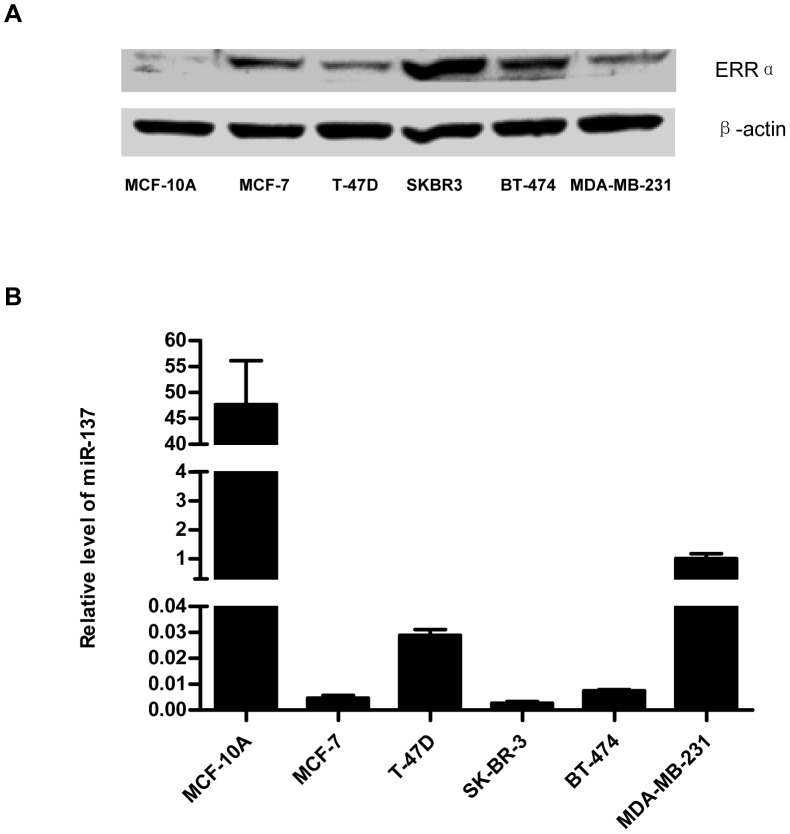
MiR-137 and ERRα levels in normal breast epithelial and breast cancer cell lines. A. Western-blot analysis for ERRα protein level in normal breast epithelial cell line (MCF-10A) and five breast cancer cell lines. β-actin was used as the loading control. B. qRT-PCR analysis for miR-137 expression level. The miR-137 expression was normalized to RNU6B-small nuclear RNA. Data are representative of three independent experiments performed in triplicate. Error bars: SD.

To establish functional association between ERRα and miR-137, we measured miR-137 and ERRα expression in normal breast epithelial cell line MCF-10A and five different breast cancer cell lines. The data showed that compared with MCF-10A, all breast cancer cell lines over-expressed ERRα (Fig. 2A) and lost the endogenous miR-137 (Fig. 2B). Furthermore, the results from available breast cancer cell lines showed that in the cell lines with relatively higher endogenous miR-137 expression (such as MDA-MB-231), a lower amount of ERRα protein was detected, whereas cell lines with lower miR-137 expression (for example SK-BR-3, BT-474 and MCF-7) showed higher amounts of the ERRα protein (Fig. 2). Although this inverse correlation between miR-137 and ERRα level was not statistically significant, it provides a possibility that the loss of miR-137 may be involved in the dysexpression of ERRα in breast tumorigenesis. Of course, as the number of available breast cancer cell lines is limited and these cultured cell lines can not stand for all subtypes of breast tumor, a more systemic study using clinical breast cancer samples is required to help us define the correlation between the endogenous expression level of ERRα and that of miR-137.

### Endogenous ERRα Expression can be Regulated by miR-137

**Figure 3:**
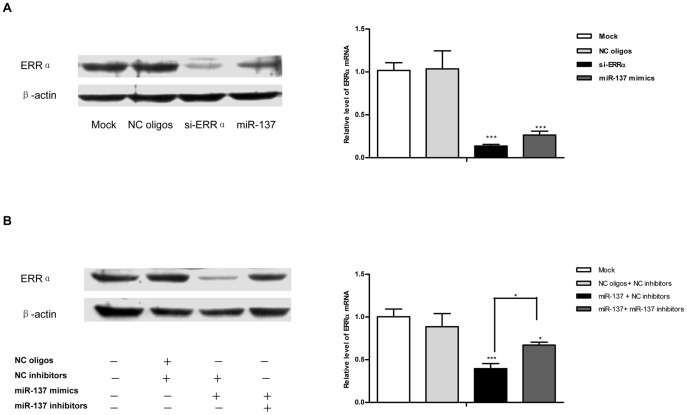
Ectopic transfection of miR-137 regulates the endogenous ERRα expression level. A. Western blot analysis for ERRα protein level and qRT-PCR analysis for ERRα mRNA level in SK-BR-3 cells 48 hr after transfection regent treatment (mock) or transfection with indicated RNA oligonucleotides (50 nM). B. Western blot analysis for ERRα protein level and qRT-PCR analysis for ERRα mRNA level in SK-BR-3 cells 48 hr after transfection regent treatment (mock) or cotransfection with equal amount of indicated RNA oligonucleotides. ERRα mRNA expression was normalized to β-actin mRNA expression. The relative level of ERRα expression determined using the 2-^△△CT^ method. Data are representative of three independent experiments performed in triplicate. Error bars: SD; *: p<0.05; ***: P<0.0001.

Given that the reporter assay showed that the 3′UTR of ESRRA contained functional miR-137 target sites, we sought to determine the effect of miR-137 mimics treatment on the regulation of the endogenous ERRα expression. As shown in Figure 3A, SK-BR-3 transfected with miR-137 mimics showed a dramatic decrease in ERRα expression at both protein level and mRNA level, compared with that of the control group. This is similar to that caused by si-ERRα transfection (Fig. 3A). Furthermore, if the SK-BR-3 cells were co-tranfected with miR-137 mimics and equal amount of specific miR-137 inhibitors, the down-regulation of ERRα expression at both protein level and mRNA level could be significantly reversed (Fig. 3B). These results demonstrate that the expression level of the endogenous ERRα can be manipulated by enforced transfection of miR-137. Herein, we should mention that although the interaction between microRNA and its target gene could induce target mRNA degradation, we can not ignore that ERRα can regulate the transcription of itself. Therefore, the down-regulation of ERRα expression at mRNA level observed by us may also be a post-effect of the decrease of the ERRα protein induced by miR-137.

### Ectopic Expression of miR-137 Inhibits Cell Proliferation and Induces Cell Cycle Arrest in Breast Cancer Cells

Recent studies employing siRNAs and synthetic antagonists have demonstrated that ERRα is required for the growth of multiple breast cancer cells in vitro or when propagated as xenografts [18], [39], [40]. Furthermore, results from functional genomic studies also showed that ERRα can directly regulate the expression of some genes associated with proliferative phenotype [17], [18]. Together, these data suggest that ERRα may be a regulator of breast tumor proliferation. Given that our data showed that miR-137 down-regulated the expression of ERRα, we hypothesized that treatment of miR-137 mimics might compromise the growth of breast cancer cells.

**Figure 4:**
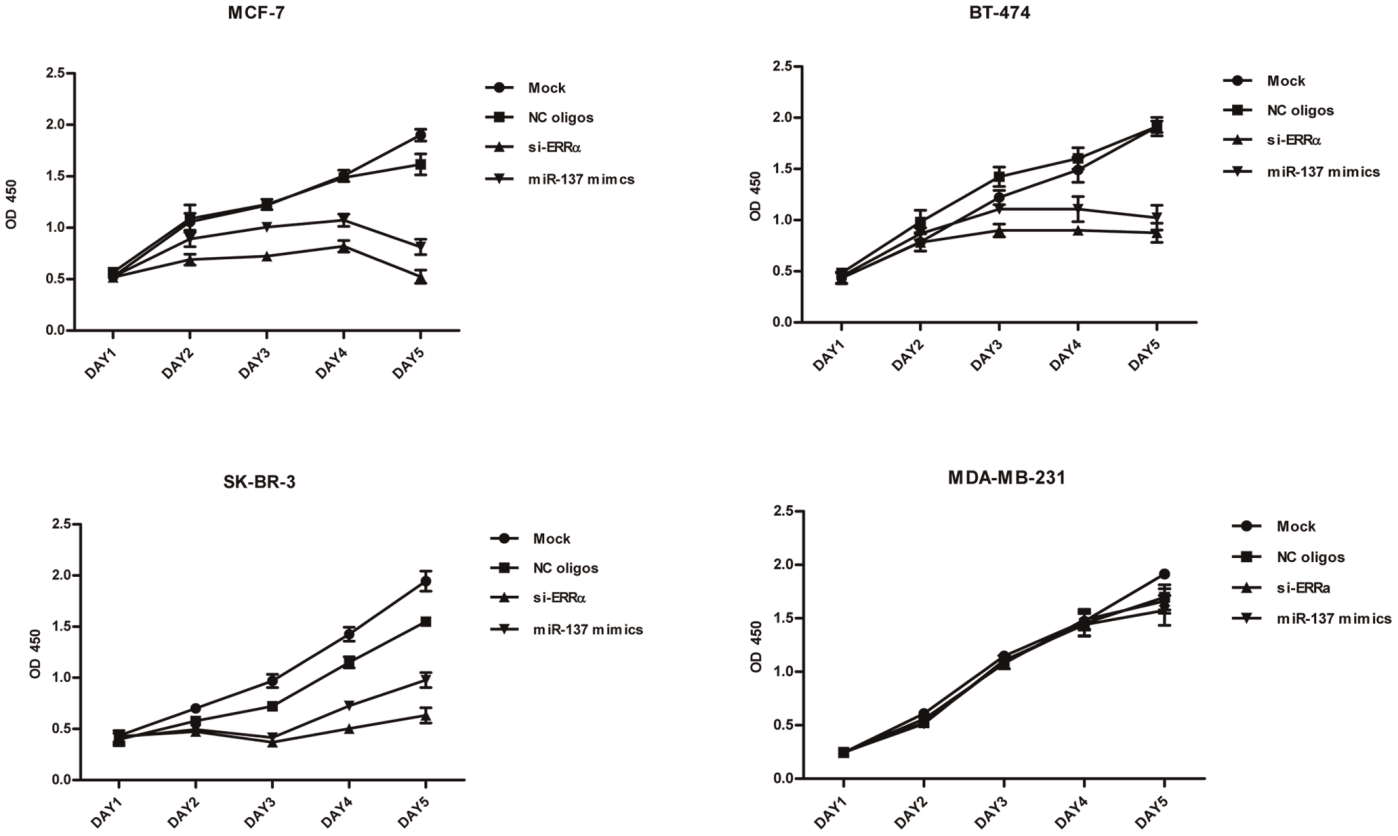
The effect of si-ERRα and miR-137–mediated knockdown of ERRα on the cell proliferation. Breast cancer cell lines (MCF-7, BT-474, SK-BR-3 and MDA-MB-231) were transfected with 50 nM NC oligos, si-ERRα or miR-137 mimics or treated with transfection reagent alone (mock) and seeded in 96-well plates. Plates were harvested at 1, 2, 3, 4, 5 days after seeding for CCK-8 assay.

Meanwhile, we also observed some papers declaring that modifying the expression of ERRα with si- or shRNA dose not impact cell proliferation in vitro [41], [42]. Thus, in order to evaluate the effect of small RNAs –mediated knockdown of ERRα on the cell proliferation, we transfected four different types of breast caner cell line with miR-137 mimics, si-ERRα and negative control oligos in parallel. The efficiency of miR-137 and si-ERRa in reducing the expression of ERRa in these cells was confirmed by western blot assay (Fig. S2). As shown in Figure 4, the silencing of ERRα significantly decreased the growth rate of breast cancer cell line MCF-7 (ERα-positive/HER2-negative), BT-474 (ERα-positive/HER2-positive) and SK-BR-3 (ERα-negative/HER2-positive), whereas, hardly influenced that of ERα-negative/HER2-negative breast cancer cell line MDA-MB-231. This phenomenon could be explained by the hypothesis that ERRα is an orphan nuclear receptor exhibiting tissue/cell-specific biological function.

**Figure 5:**
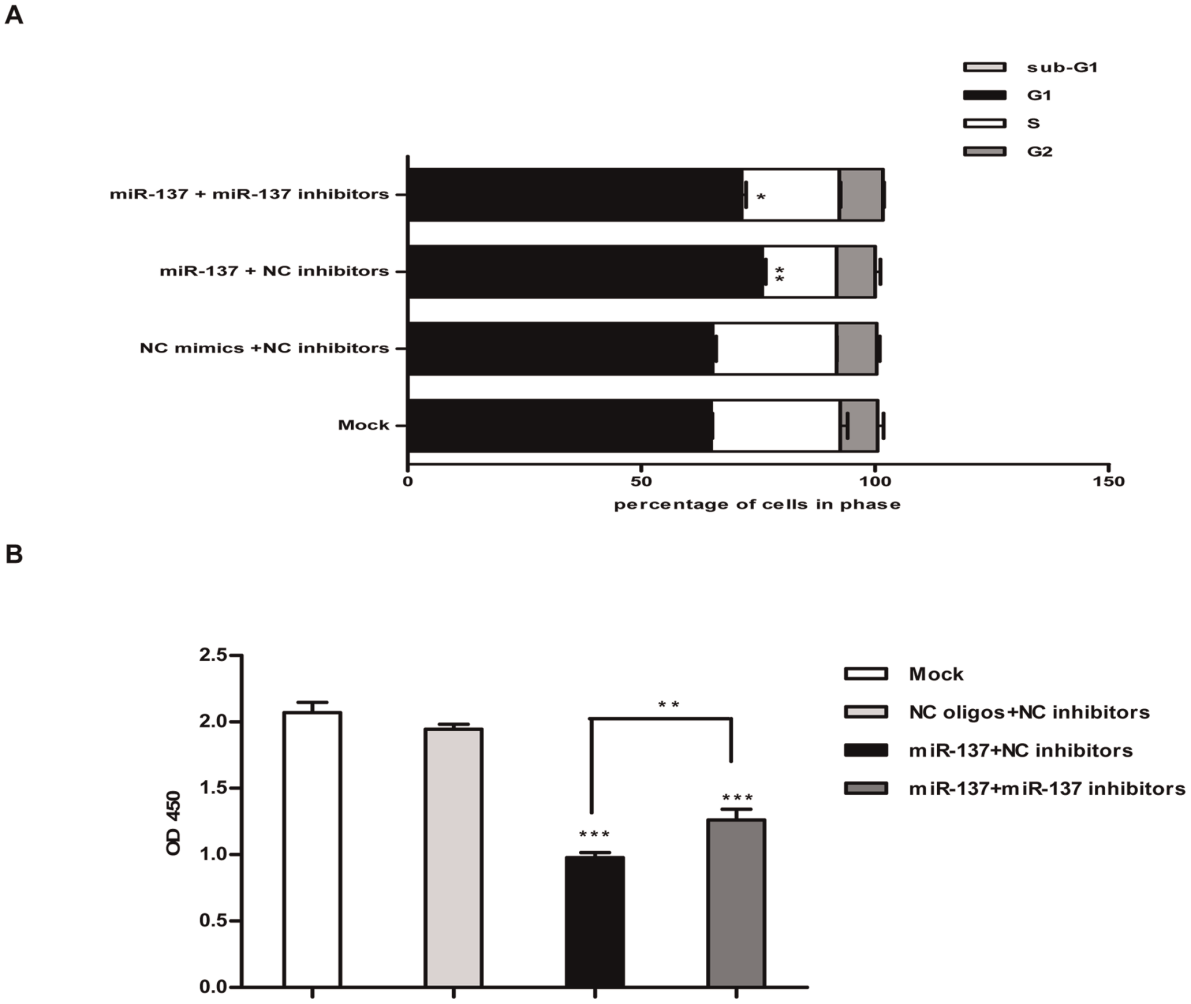
MiR-137 induces cell cycle G1 arrest in SK-BR-3 cells. A. Cell cycle analysis in SK-BR-3 cells transfected with indicated RNA oligonucleotides for three days using propidium iodide staining and flow cytometry. The percentage of cells in each cell cycle phases was quantified. Data are representative of three independent experiments performed in duplicate. Error bars: SD; *: p<0.05; **: P<0.01. B. BrdU incorporation assay performed in SK-BR-3 cells transfected with indicated RNA oligonucleotides for three days. Data are representative of three independent experiments performed in triplicate. Error bars: SD; **: P<0.01; ***: P<0.0001.

Since the SK-BR-3 cell line is generally considered as a cellular model of breast cancer exhibiting high ERRα activity and is sensitive to growth inhibition by ERRα depletion or inactivation [39], we further investigated the detailed mechanisms underlying the inhibition of cell proliferation mediated by miR-137 in this cell line. Analysis of cell cycle phase distribution by cytometry showed that compared with negative control group, the cell cycle progression of SK-BR-3 cells transfected with miR-137 mimics were arrested at G1 phase with a significant decrease in S and G2 phase. While the miR-137 mimics were “neutralized” by the co-transfected miR-137 inhibitors, the percentage of G1 phase decreased, and the other phases increased accordingly, suggesting that cell cycle G1 phase arrest was partly reversed (Fig. 5A). Furthermore, the absence of a sub-G1 cell population was detected by flow cytometry, suggesting that the transfection of miR-137 does not lead to cell apoptosis (Fig. 5A). In addition, we also observed the effect of miR-137 on cell cycle progression by BrdU incorporation assay. As shown in Figure 5B, after transfection of miR-137, the number of cells in cell cycle S phase decreased significantly. Taken together, these data indicate that the ectopic expression of miR-137 can trigger cell proliferation inhibition through arresting cell cycle at G1 phase.

### MiR-137 Influences Cell Proliferation Partly through Regulating the Expression of ERRα Downstream Target Gene-cell Cycle Protein CyclinE1 (CCNE1)

**Figure 6:**
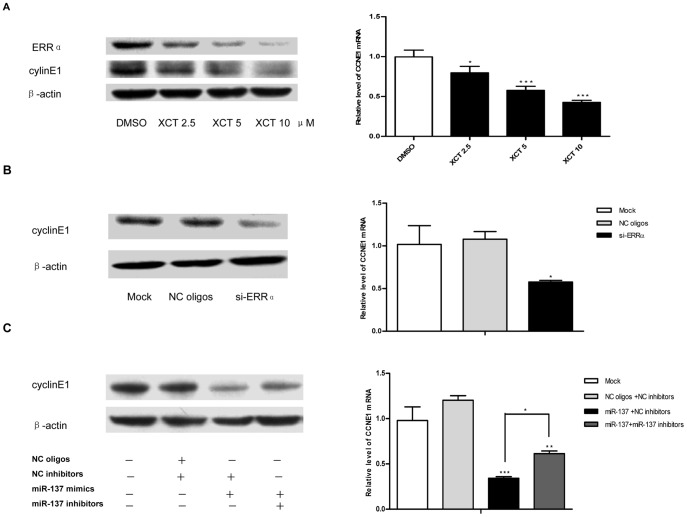
The expression of ERRα downstream target gene CCNE1 is regulated by miR-137. A. Western blot analysis for ERRα and CylinE1 protein level and qRT-PCR analysis for CCNE1 mRNA level in SK-BR-3 cells 48 hr after DMSO or XCT-790 treatment. B. Western blot analysis for CylinE1 protein level and qRT-PCR analysis for CCNE1 mRNA level in SK-BR-3 cells 48 hr after transfection regent treatment (mock) or transfection with NC oligos or si-ERRα. C. Western blot analysis for CylinE1 protein level and qRT-PCR analysis for CCNE1 mRNA level in SK-BR-3 48 hr after transfection regent treatment (mock) or co-transfection with equal amount of indicated RNA oligonucleotides. CCNE1 mRNA expression was normalized to β-actin mRNA expression. The relative level of CCNE1 mRNA was determined using the 2-^△△CT^ method. Data are representative of three independent experiments performed in triplicate. Error bars: SD; *: p<0.05; **: P<0.01; ***: P<0.0001.

Given that our study suggested that depletion of ERRα by miR-137 could impair the cell cycle progression, we wondered which ERRα-regulated pathways might contribute to this effect. According to the result of genome-wide identification of direct target genes of ERRα in breast cancer cell lines, cell cycle protein cyclinE1 (CCNE1), which regulates the progression of cell cycle from G1 to S phase, could be a direct target gene of ERRα [17]. As an initial step in our analysis, we demonstrated that in SK-BR-3 cells, the expression of CCNE1 was indeed under the control of ERRα. As shown in Figure 6A, treatment with the specific inverse agonist XCT-790 resulted in the dose-dependent inhibition of CCNE1 expression at both transcriptional and protein levels. Furthermore, the knock-down of ERRα by si-ERRα exhibited similar effect on the CCNE1 expression (Fig. 6B). We then evaluated the expression of CCNE1 in SK-BR-3 cells following the treatment of miR-137 mimics. Not surprisingly, a markedly decrease of CCNE1 expression at both mRNA level and protein level was observed in the SK-BR-3 cells transfected with miR-137 mimics. Moreover, this effect was reversed by the existence of specific miR-137 inhibitors (Fig. 6C), suggesting that miR-137 mimics has the effect on the regulation of CCNE1 expression.

**Figure 7:**
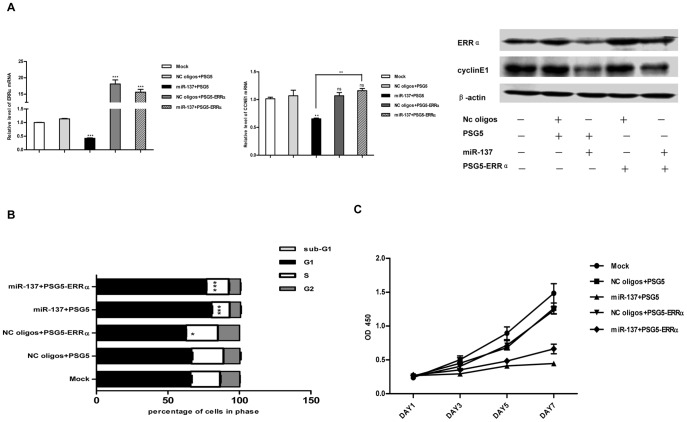
MiR-137 influences cell proliferation in SK-BR-3 cells partly through ERRα-CCNE1 axis. A. re-expression of ERRα (without 3′-UTR) in SK-BR-3 cells transfected with miR-137 reversed the decrease of CCNE1 expression induced by miR-137. qRT-PCR analysis for ERRα and CCNE1 mRNA level and western blot analysis for ERRα and CylinE1 protein level in SK-BR-3 cells 48 hr after transfection. CCNE1 or ERRα mRNA expression was normalized to β-actin mRNA expression. The relative level of CCNE1 or ERRα determined using the 2-^△△CT^ method. Data are representative of three independent experiments performed in duplicate. Error bars: SD; **: P<0.01; ***: P<0.0001. B. re-expression of ERRα (without 3′-UTR) in SK-BR-3 cells transfected with miR-137 partly rescued the arrested cell cycle progression. Cell cycle analysis using propidium iodide staining and flow cytometry was performed in SK-BR-3 cells transfected with indicated RNA oligonucleotides (50 nM) and plasmids (300 ng) for three days. The percentage of cells in each cell cycle phases was quantified. Error bars: SD; *: p<0.05; ***: P<0.0001. C. re-expression of ERRα (without 3′-UTR) in SK-BR-3 cells transfected with miR-137 partly rescued the impaired proliferation capacity. SK-BR-3 cells were transfected with indicated RNA oligonucleotides (50 nM) and plasmids (30ng) and seeded in 96-well plates. Plates were harvested at 1, 3, 5, 7 days after seeding. Cell numbers were determined by CCK-8 assay.

In order to demonstrate that miR-137 acts on the regulation of CCNE1 expression and cell cycle progression through ERRα, we tested whether exogenously expressed ERRα (without 3′-UTR) could restore the reduced CCNE1 expression and impaired proliferative phenotype in SK-BR-3. In cells treated with NC oligos, overexpression of ERRα failed to significantly increase the expression of CCNE1 or promote the cell proliferation (Fig. 7), probably due to a sufficiently high endogenous level of ERRα already existing in SK-BR-3 cells. However, ectopic transfection with plasmid encoding ERRα without 3′-UTR robustly reversed the decreased expression of CCNE1 induced by miR-137 at both transcriptional and protein levels (Fig. 7A), and partly restored the arrested proliferation (Fig. 7B and 7C). Together, all of these data indicate that miR-137 induces cell cycle G1 phase arrest and cell proliferation suppression, at least in part, via the ERRα–cyclinE1 pathway.

### MiR-137 Influences the Migratory Capacity of MDA-MB-231 Partly through ERRα-WNT11 Signaling Pathway

**Figure 8:**
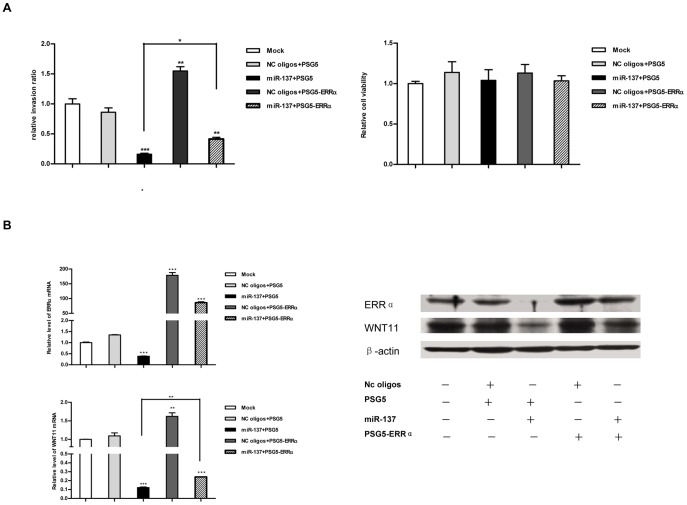
MiR-137 influences the migratory capacity of MDA-MB-231 cells partly through ERRα-WNT11 signaling pathway. A. re-expression of ERRα (without 3′-UTR) in MDA-MB-231 cells restored the impaired migratory capacity induced by miR-137. MDA-MB-231 cells were co-transfected with indicated RNA oligonucleotides (50 nM) and plasmids (1 µg), and serum starved for 12 hr, followed by assessment of cell invasion and viability. Error bars: SD; *: p<0.05; **: P<0.01; ***: P<0.0001. B. re-expression of ERRα (without 3′-UTR) in MDA-MB-231 cells reversed the decrease of WNT11 expression induced by miR-137. MDA-MB-231 cells were co-transfected with indicated RNA oligonucleotides (50 nM) and plasmids (1 µg). 48 hr after transfection, protein and mRNA levels of WNT11 and ERRα were assayed using western bolt and qRT-PCR respectively. WNT11 or ERRα mRNA expression was normalized to β-actin mRNA expression. The relative level of WNT11 or ERRα determined using the 2-^△△CT^ method. Data are representative of three independent experiments performed in duplicate. Error bars: SD; **: P<0.01; ***: P<0.0001.

In addition to its role in the regulation of cancer cell proliferation, ERRα has been implicated in promoting cancer cell migration [18], [43]. MDA-MB-231 is a breast cancer cell line with high migratory capacity. In our study, we did not observe the significant inhibition of growth in MDA-MB-231 treated with miR-137 mimics (Fig. 4) but we found that treatment of miR-137 led to dramatic decrease in migration/invasion of MDA-MB-231 (Fig. 8A), which is consistent with the previous study that knockdown of ERRα by si-ERRα in MDA-MB-231 had no effect on in vitro cell proliferation but reduced the migratory potential of these cells [18].

Therefore, we next sought to illustrate the mechanism through which miR-137 inhibits the migration of MDA-MB-231. Given that WNT11 has been considered as a key mediator of the promigratory activity of ERRα/β-cat complex in several cancer cell lines including MDA-MB-231 [43], we tested the effect of miR-137 on regulating the expression of WNT11. As shown in Figure 8B, miR-137 exhibited high efficacy in reducing the expression of endogenous ERRα and its target gene WNT11 at both transcriptional and protein levels in MDA-MB-231. More importantly, the reduced expression of WNT11 was partly restored by the ectopic expression of ERRα without 3′-UTR. Furthermore, we also observed that the re-expression of ERRα deleted from its 3′-UTR significantly restored the migratory capacity impaired by miR-137 mimics. Meanwhile, our data also showed that the experimental treatment did not influence the viability of treated cells (Fig. 8A). Together, these data suggest that miR-137 decreases the migration/invasion of MDA-MB-231 partly through ERRα-WNT11 pathway.

## Discussion

Increasing evidences in the past few years, especially the high throughput functional genomic studies have demonstrated that ERRα is an orphan nuclear receptor that plays important roles in breast cancer progression and the heterogeneity of the disease [44]. To further understand the contribution of ERRα to breast cancer progression, it is essential to better define the detailed regulatory mechanism of ERRα expression. However, the mechanisms underlying the dysexpression of this nuclear receptor in breast cancer remain to be investigated.

As microRNAs are very important regulators of gene expression and so far there is no report about the regulation of ERRα by any microRNA, we sough to determine whether the expression of ERRα is under the control of microRNAs. Through biochemical experiment we demonstrated that miR-137 significantly down-regulated the expression of ERRα in breast cancer cells through recognizing two highly-conserved miR-137 target sites located in the 3′-UTR of ERRα. MiR-137 is located on chromosome 1p22, a region embedded in a CpG island. Therefore, this miRNA has been found to be frequently silenced by methylation in several cancers including colorectal cancer [45], gastric cancer [46], uveal melanoma [47], oral cancer [48], glioblastoma multiforme [49] and squamous cell carcinoma of the head and neck [50], [51], and potentially acts as a tumor suppressor microRNA in these tumors. However, the expression level and downstream target genes of miR-137 as well as its biological roles in breast cancer are still unknown. In our study, we found that compared with that of normal breast epithelial cell line (MCF-10A), the expression level of miR-137 was also dramatically decreased in different breast cancer cell lines. Furthermore, there seemed to be an inverse association between the miR-137 level and the ERRα expression in the breast cancer cell line we tested, which suggests that the silencing of miR-137 in tumor cells, especially in breast cancer, may be involved in the dysregulation of ERRα and contributed to breast tumorigenesis.

Based on the finding that miR-137 regulates the expression of ERRα, we further investigated the functional consequence of this effect. In the past few years, a large number of studies made efforts to elucidate the direct effect of ERRα in breast tumor biology [42]. Although results from functional genomic studies showed that a large number of ERRα target genes are associated with cell metabolism [17], [18], whether and how its role as metabolic regulator is involved in the pathophysiology of cancer remains to be addressed. Moreover, some reports have shown that, in tumor cells, ERRα exert other effects aside from the activity of metabolic control, such as the direct regulation of tumor proliferation and migration [39], [43]. Therefore, we focused our study on the effect of miR-137 on modulating the proliferative and migratory capacity of breast cancer cell lines. In our studies, we observed that the knock –down of ERRα by either si-ERRα or miR-137 impaired the proliferation of breast cancer cell lines we tested except that of MDA-MB-231. For MDA-MB-231, silencing of ERRα had little impact on the cell growth but dramatically inhibited its migratory capacity. This kind of cell-specific consequence of loss of ERRα may result from the cell-specific biological function of the nuclear receptor. ERRα is an orphan nuclear receptor whose biological effect dependents on the combination with various co-regulators, which suggests that in different molecular environment, ERRα may exert different functions. Given the complexity of molecular environment of different breast cancer cell lines, we took SK-BR-3 (a breast cancer cell line that is sensitive to growth inhibition induced by depletion of ERRα) and MDA-MB-231 (a breast cancer cell line with high migratory capacity) as cell models respectively to further elucidate the mechanism underlying the inhibitory effect of miR-137 on the proliferation and migration of breast cancer cells. Of course, we also realized that the functional effects of miR-137 treatment we observed here were not solely the consequence of the change of the ERRα level. Actually, besides ERRα, some other genes (such as Cdc42 [52]) associated with cell proliferative and migratory phenotypes are also the targets of miR-137. Therefore, we took the rescue experiment using ERRα (3′-UTR deleted) re-expression to evaluate the contribution of ERRα to these effects. Our data suggest that down-regulation of ERRα expression is at least one component of the mechanisms underlying the tumor-suppressing effect of miR-137 in breast cancer.

In the present study, we also tested the expression of some tumorigenesis-related target genes of ERRα after miR-137 treatment. Among them, two identified ERRα direct down-stream target genes: CCNE1 and WNT11 are of particular interest to us. CyclinE1 is not only an important cell cycle regulator, but also an independent prognostic marker of breast cancer [53], [54]. The gene CCNE1 was found to be amplified in about 12% of invasive breast tumor patients [55]. Furthermore, the latest study showed that the overexpression of CCNE1 in HER2 positive tumor can impair the anti-HER2 therapy through resulting in resistance to trastuzumab both in vitro and in vivo, whereas the mechanisms leading to CCNE1 over-expression in these cells are unclear [56]. Our study validated the existence of activated ERRα-CCNE1 signaling pathway in HER2-positive breast cancer cell line–SK-BR-3, which suggests that the dysexpression of ERRα may be one of the factors contributing to the over-expression of CCNE1 in breast tumor. As miR-137 interferes with the ERRα-CCNE1 axis, its role and therapeutic value in breast cancer, especially in the HER2 positive breast cancer are worth further investigation. WNT11 has been found upregulated in several cancers [57], and its expression has been previously associated with increased cell migration [58]. Recent study demonstrated that WNT11 expression is directly co-regulated by ERRα and β-catenin in several cancer cells, which is considered as the key mechanism underlying the promigratory activity of ERRα [43]. In the present study, we demonstrated that miR-137 decreased the migration/invasion of MDA-MB-231 partly through ERRα-WNT11 pathway, providing an alternative way to inhibit the migration of cancer cells with high migratory capacity.

In addition, we also noticed that the ectopic expression of miR-137 did not interfere with all of the ERRα signaling pathways. Although miR-137 indeed changes the expression of some ERRα target genes, such as ACO2 (Fig. S3) and the two genes we mentioned above, the expression of other identified classic ERRα target genes including HER2 (ERBB2) (Fig. S3) and VEGF (data not shown) seems not to be affected by miR-137. To our knowledge, a reasonable explanation to this phenomenon may be the complexity of the gene transcriptional regulation. Usually, the transcription of a certain gene is under the control of multiple transcriptional factors or cofactors, and the alteration of the expression level is a result of the dynamic balance of each component in this complex. Moreover, as it was mentioned above, one certain microRNA can also target more than one molecule. This means ERRα is not the only transcriptional factor targeted by miR-137 in the same cell.

In conclusion, our studies establish a role of miR-137, a microRNA having potential tumor suppressor activity, in negatively regulating the expression of ERRα, a nuclear receptor considered to facilitate the progression of breast cancer, and in inhibiting the proliferative and migratory phenotype of some breast cancer cells. These results expand our understanding of the mechanism underlying the regulation of ERRα expression and suggest that the aberrant expression of miR-137 may be involved in breast cancer progression. The ectopic expression of miR-137 may serve as a useful tool in manipulating the expression level of ERRα and exploring the function of ERRα in breast cancer.

## Materials and Methods

### RNA Oligonucleotides

Has-miR-137 mimics, has-miR-137 inhibitors, siRNA against ERR α (sense: 5′- AGAGGAGUAUGUUCUACUAAAGGCC-3′) negative control (NC) oligos (sense: 5′-UUCUCCGAACGUGUCACGUTT-3′) and microRNA inhibitor negative control (NC): (5′-CAGUACUUUUGUGUAGUACAA-3′) were chemically synthesized by GenePharma (Shanghai, China).

### Cell Culture

Human breast cancer cell lines BT-474, T-47D and MDA-MB-231 were cultured in RPMI-1640 medium (Gibco, Shanghai, China) with 10% FBS (Gibco, Gaithersburg, USA), 1% NEAA (Hyclone, Logan, Utah, USA), 1% sodium pyruvate (Hyclone) and 1% penicillin-streptomycin (Gibco, Gaithersburg, USA). Human breast cancer cell lines MCF-7 and SK-BR-3 were cultured in MEM medium (Gibco, Shanghai, China) and Mccoy’s medium (Gibco, Gaithersburg, USA) with 10% FBS, 1% NEAA, 1% sodium pyruvate and 1% penicillin-streptomycin respectively. The human breast epithelial cell line MCF-10A was cultured in DMEM/F-12 (Hyclone) with 5% horse serum (MinHai Bio-engineering, Lanzhou, China), 10 ug/ml insulin (Sigma-Aldrich, Saint Louis, MO, USA), 20 ng/ml EGF (Sigma-Aldrich), 100ng/ml cholera toxin (Calbiochem, Darmstadt, Germany), 0.5 ug/ml hydrocortisone (Sigma-Aldrich) and 1% penicillin-streptomycin. The human liver hepatocellular carcinoma cell line HepG2 was cultured in DMEM medium (Gibco, Shanghai, China) with 10% FBS, 1% NEAA, 1% sodium pyruvate and 1% penicillin-streptomycin. All cultured cell lines were purchased from ATCC.

### Luciferase Reporter Plasmids Construction

To construct the reporter plasmids contain consensus or mismatch miR-137 target site, oligonucleotide pairs that contain the desired miR-137 target region and restriction enzymes sites (N*he Ι* and S*al ΙΙ*) were annealed and ligated into the Firefly-Renilla dual reporter vector-pmirGLO Vector (Promega, Madison, WI, USA). For construction of reporter plasmids with wide type or mutant ESRRA3’UTR, total RNA from SK-BR-3 cells was reverse transcribed to the first strand of cDNA by SuperScript III kit (Invitrogen, Carisbad, CA, USA) with the primer oligo (dT)_18_ (Takara, Dalian, China). The 3′ UTR of the human ERRα gene (NM_004451) was amplified by PCR with the cDNA of SK-BR-3 cells as template. Purified PCR products were inserted downstream of the firefly luciferase gene in the Firefly-Renilla dual reporter vector- pmirGLO-vector after digested by N*he Ι* and S*al ΙΙ* (Takara). The construct was designated as WT 3′UTR. The deletion mutated 3′UTR were amplified by PCR with WT3’UTR as the template using the site-directed mutagenesis kit (Takara), inserted into the same reporter vector and named mutant A, mutant B and mutant C, respectively. The sequences of primers used for luciferase reporter plasmids construction were shown in Table 1.

**Table 1:** Primers used for PCR.

Primer name	Sequence
WT 3′UTR Forward	5′- AGAGCTAGCGGCAAGGGGTGGGACTG-3′
WT 3′UTR Reverse	5′- CGCGTCGACGAGCTCGGTATTATATAT-3′
Mutant A Forward	5′-CTCCAAGCAGACTCCAGCCCCTGGAC-3′
Mutant A Reverse	5′-TCAAGGGGGGACACTAATGCCCAATG-3′
Mutant B Forward	5′-CACTATATTTATTTTTGGGTTTGGCCAGGG-3′
Mutant B Reverse	5′-CTGCAGGGCATAGAGGCAGTGCTCTC-3′
Q-ESRRA Forward	5′-GGCCCTTGCCAATTCAGA-3′
Q-ESRRA Reverse	5′-GGCCTCGTGCAGAGCTTCT-3′
Q-CCNE1 Forward	5′-CTGGACAAAGCCCGAGCAAAG-3′
Q-CCNE1 Reverse	5′-CCTCCGCTGCAACAGACAGAA-3′
Q-WNT11 Forward	5′-GCTTGTGCTTTGCCTTCACTTGGA-3′
Q-WNT11 Reverse	5′-TGGCCCTGAAAGGTCAAGTCTGTA-3′
Q-ACO2 Forward	5′-GATCCACGAGACCAACCTGAAGAA-3′
Q-ACO2 Reverse	5′-CCTTCATTCTGTTGAGGGCACTGC-3′
Q-β-actin Forward	5′-CACCAACTGGGACGACAT-3′
Q-β-actin Reverse	5′-GCACAGCCTGGATAGCAAC-3

The recognition sites of restriction endonuclease are underlined.

### Transfection

RNA oligonucleotides were transfected into cells using Lipofectamine RNAiMAX (Invitrogen) according to the manufacture’s protocol of reverse transfection. RNA oligonucleotides and plasmids for rescue experiment are co-transfected into cells using instantFECT according to the manufacture’s protocol of reverse transfection.

### Luciferase Assay

Cells were seeded in 24-well plate with regular growth medium without antibiotics 1 day before transfection and transiently co-transfected with the reporter plasmid (150 ng/well) and miR-137 mimics or its NC oligos at the concentration of 20 nM/well using lipofectamime 2000 (Invitrogen). Twenty-four hours after transfection, cell lysates were collected and luciferase activities were measured by a Dual-Luciferase Reporter System (Promega) using TD 20/20 luminometer (Promega) following the manufacturer’s protocol. The luminescence intensity of Firefly luciferase was normalized to that of Renilla luciferase.

### ERRα Inverse Agonist XCT-790 Treatment

Cells were seeded in 6-well plate. The following day, change the medium with fresh medium with DMSO (Sigma-Aldrich) or XCT-790 (Sigma-Aldrich) at a final concentration of 2.5 µM, 5 µM, 10 µM respectively. Cells were harvested 48 hr after treatment for RNA or protein extraction.

### Endogenous miR-137 Expression Assay by Real-time RT-PCR

Total RNAs, including total miRNAs, were isolated from cultured cell lines using Qiazol and miRNeasy Mini kit (QIAGEN, Maryland, USA), according to the manufacture’s instructions. The RT reactions were performed according to TaqMan® small RNA Assay protocol by using commercial small RNA primers of has-miR-137 or RNU6B in a 15 µl volume with 10 ng total RNA. Real-time PCR was performed using ABI 7900 HT system in a 96 well plate format. All reagents were purchased from Applied Biosystems, Inc. (Foster City, CA). The expression levels of mature miR-137 were normalized relative to RNU6B-small nuclear RNA, the fold change in miR-137 expression was obtained using the2-^△△CT^ method.

### mRNA Expression Assay by Quantitative PCR Analysis

Total RNA was extracted using Trizol (Invitrogen) and reverse-transcribed into cDNA with reverse transcriptase M-MLV (Invitrogen) following the manufacturer’s manual. Quantitative-Real-time PCR was performed using IQ™ SYBR Green Supermix (Bio-Rad, Hercules, CA, USA) on the iQ-5 Real-time PCR Detection System (Bio-Rad). Expression level of ERRα, CCNE1 and WNT11 mRNA were normalized to β-actin mRNA. The relative level of mRNA was determined using the 2-^△△CT^ method. The sequences of primers used for quantitative PCR analysis were shown in Table 1.

### Western Blot

Total cell lysates were prepared using protein lysis buffer (150 mM NaCl, 1% NP-40, 50 mM Tris-HCl (pH 7.4), 1 mM EDTA) containing a cocktail of protease inhibitor (Roche, Shanghai, China). Equal amounts of protein sample (40–50 µg) was separated by 8% SDS-PAGE and transferred to PVDF membrane (Millipore, Bedford, MA, USA) using the Bio-Rad semi-dry transfer system. After 1 hr blocking in 1×Tris-buffered saline with 5% non-fat milk at 37°C, the membrane was immunoblotted overnight at 4°C with primary antibody. The antibodies used were as follows: anti-ERRα rabbit polyclonal antibody (1∶500, Millipore, cat. #07-662), anti-CyclinE1 rabbit monoclonal antibody (1∶800, Millipore cat. #04-222), anti-WNT11 rabbit polyclonal antibody (1∶600, abcam, cat.ab31962),anti-β-actin mouse monoclonal antibody (1∶500, Santa Cruz, California, USA cat. sc-47778). Horseradish peroxidase labeled goat anti-mouse or goat anti-rabbit secondary antibody (Zsbio, Shanghai, China) was incubated with the membrane at a concentration of 1∶30000 for 1 hr at 37°C. After washing with 1×TBST, signals were detected by incubating membrane with ECL reagent from Thermo (Rockford, IL, USA) and exposing it to an x-ray film and developed.

### Cell Proliferation Assay

To measure cell proliferation, cells were seeded at a density of 2000-7000 cells per well into 96-well plates at Day0 (initiate from small RNAs or plasmids transfection). The plates were harvested for Cell Counting Kit-8 (Dojindo Laboratories Kumamoto, Japan) measurement at indicated time point. The OD values at 450 nM were measured using the multiskan spectrum (Thermo) with SkanIt software 2.2.

### Cell Cycle Assay

Cells were trypsinized 72 h after transfection. Cell pellets harvested by centrifugation were washed for twice with ice-cold PBS and fixed with ice-cold 70% ethanol for 48 hr at 4°C. Staining for DNA content was performed with 50 mg/ml propidium iodide (Sigma-Aldrich) and 1 mg/ml RNase A (Sigma-Aldrich) for 30 min. Cell cycle analysis was performed with a million cells in each group on FACSCalibur™ (BD Bioscience) with Muticycle for Windows software (Beckman coulter).

### BrdU Incorporation Assay

SK-BR-3 cells were seeded at a density of 2000 cells per well into 96-well plate at Day0 (initiate from miR-137 mimics and miR-137 inhibitors transfection). At day3, the cells were harvested for BrdU enzyme-linked immunosorbent assay (cell signaling) following the manufacturer’s instructions.

### Transwell Assay

Forty eight hours after transfection, 2.5×10^4^ MDA-MB-231 cells were suspended in 500 µL serum free culture medium and plated in duplicate in the top chamber with matrigel-coated membrane (24-well insert; pore size 8 µm; Becton Dickinson). The cells migrated toward culture medium with 10% FBS for 16 hours, after which the cells did not invade were removed. Cells on the lower surface of the membrane were stained in 5% crystal violet in 20% methanol and counted under microscope. Cell viability assays were performed in parallel with the invasion assay using the Cell Counting Kit-8. Cells (2.5×10^4^ cells in 100 µL medium) were seeded in quadruplicate on a 96-well plate for 16 hours followed by the addition of CCK8 for 1 hour. The OD values at 450 nM were measured using the multiskan spectrum with SkanIt software 2.2 (Thermo), and the relative cell viability was quantified.

### Statistical Analysis

Results are displayed as the mean±SD from duplicate or triplicate samples for each group. Significant differences were established by either the Student’s t-test or one-way ANOVA, according to the number of groups compared, using the computer program GraphPad Prism (GraphPad Software Inc V4.03, San Diego, CA, USA). When significant variations were found by one-way ANOVA, the Tukey-Kramer multiple comparisons post-test was performed.

## Supporting Information

Figure S1 **The HepG2 cells express relatively lower level of endogenous ERRα.** Western-blot analysis for ERRα protein level in three breast cancer cell lines (BT-474, MCF-7 and SK-BR-3) and human liver hepatocellular carcinoma cell line HepG2. β-actin was used as the loading control.
(TIF)

Figure S2 **Sliencing of ERRα by si-ERRα or miR-137 mimics in MCF-7, BT-474 and MDA-MB-231 cell line.** Western blot analysis for ERRα protein level in MCF-7 (A), BT-474 (B) and MDA-MB-231 (C) cells 48 hr after transfection regent treatment (mock) or transfection with NC oligos, si-ERRα or miR-137 mimics. β-actin was used as the loading control.
(TIF)

Figure S3 **The effect of miR-137 treatment on ACO2 and ERBB2 expression.** A. qRT-PCR analysis for ACO2 level in SK-BR-3 cells 48 hr after transfection with NC-oligos, si-ERRα or miR-137 mimics. B. qRT-PCR analysis for ERBB2 level in SK-BR-3 cells 48 hr after transfection with NC-oligos, si-ERRα or miR-137 mimics. ACO2 and ERBB2 expression was normalized to β-actin mRNA expression. The relative expression level of was determined using the 2-^△△CT^ method. Data are representative of three independent experiments performed in triplicate. Error bars: SD; *: p<0.05; **: p<0.01.
(TIF)
