# Supplementary figures and images for: MiR-137 Targets Estrogen-Related Receptor Alpha and Impairs the Proliferative and Migratory Capacity of Breast Cancer Cells
Source: PLoS One. 2012 Jun 18;7(6):e39102. doi: 10.1371/journal.pone.0039102 (PMC3377602; doi:10.1371/journal.pone.0039102)

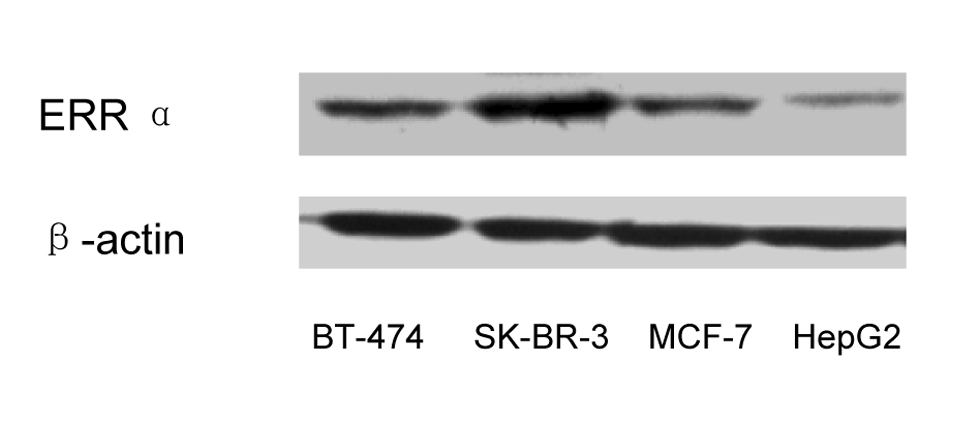

Supplement: Figure S1 — The HepG2 cells express relatively lower level of endogenous ERRα. Western-blot analysis for ERRα protein level in three breast cancer cell lines (BT-474, MCF-7 and SK-BR-3) and human liver hepatocellular carcinoma cell line HepG2. β-actin was used as the loading control. (TIF) [file pone.0039102.s001.tif]

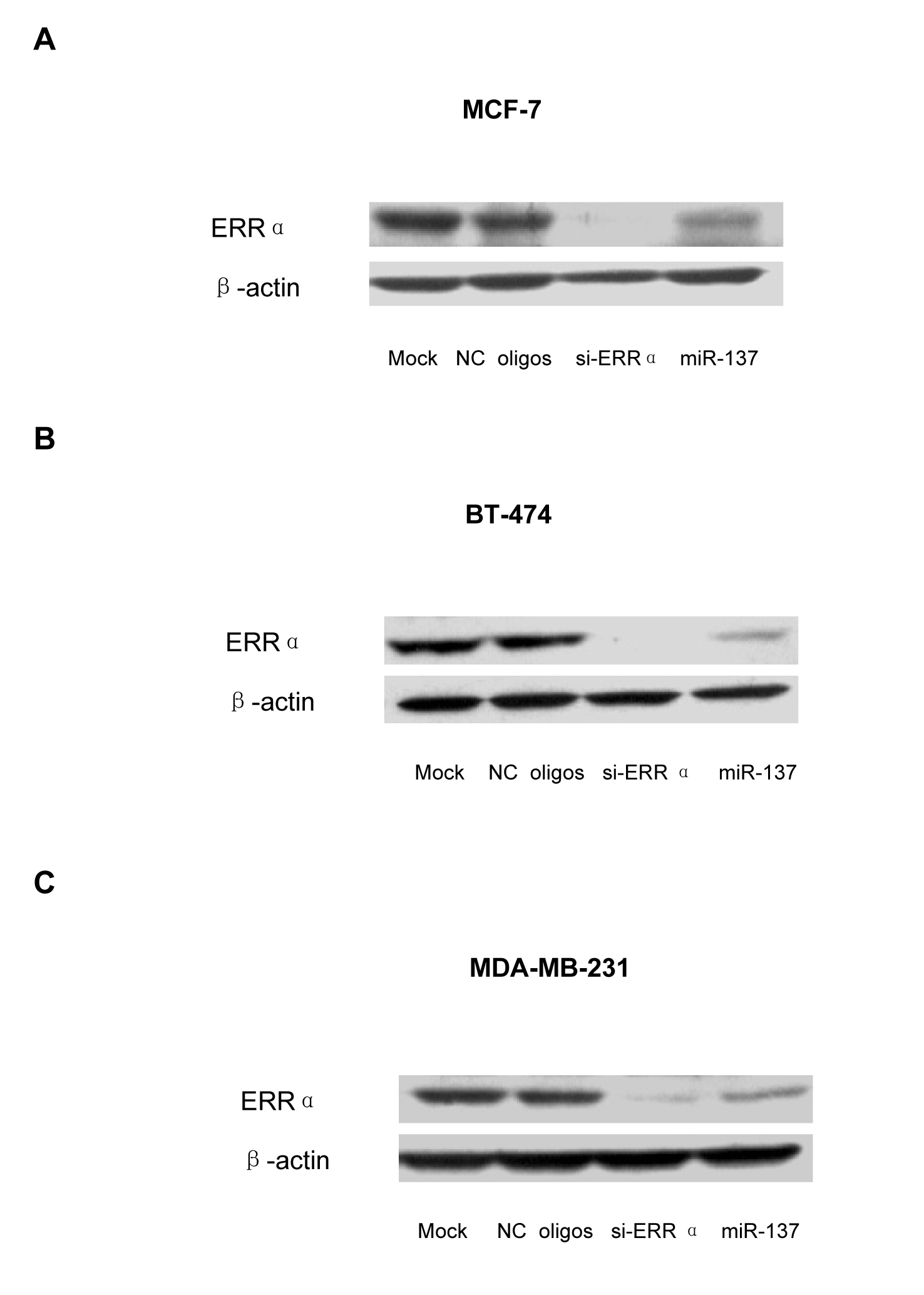

Supplement: Figure S2 — Sliencing of ERRα by si-ERRα or miR-137 mimics in MCF-7, BT-474 and MDA-MB-231 cell line. Western blot analysis for ERRα protein level in MCF-7 (A), BT-474 (B) and MDA-MB-231 (C) cells 48 hr after transfection regent treatment (mock) or transfection with NC oligos, si-ERRα or miR-137 mimics. β-actin was used as the loading control. (TIF) [file pone.0039102.s002.tif]

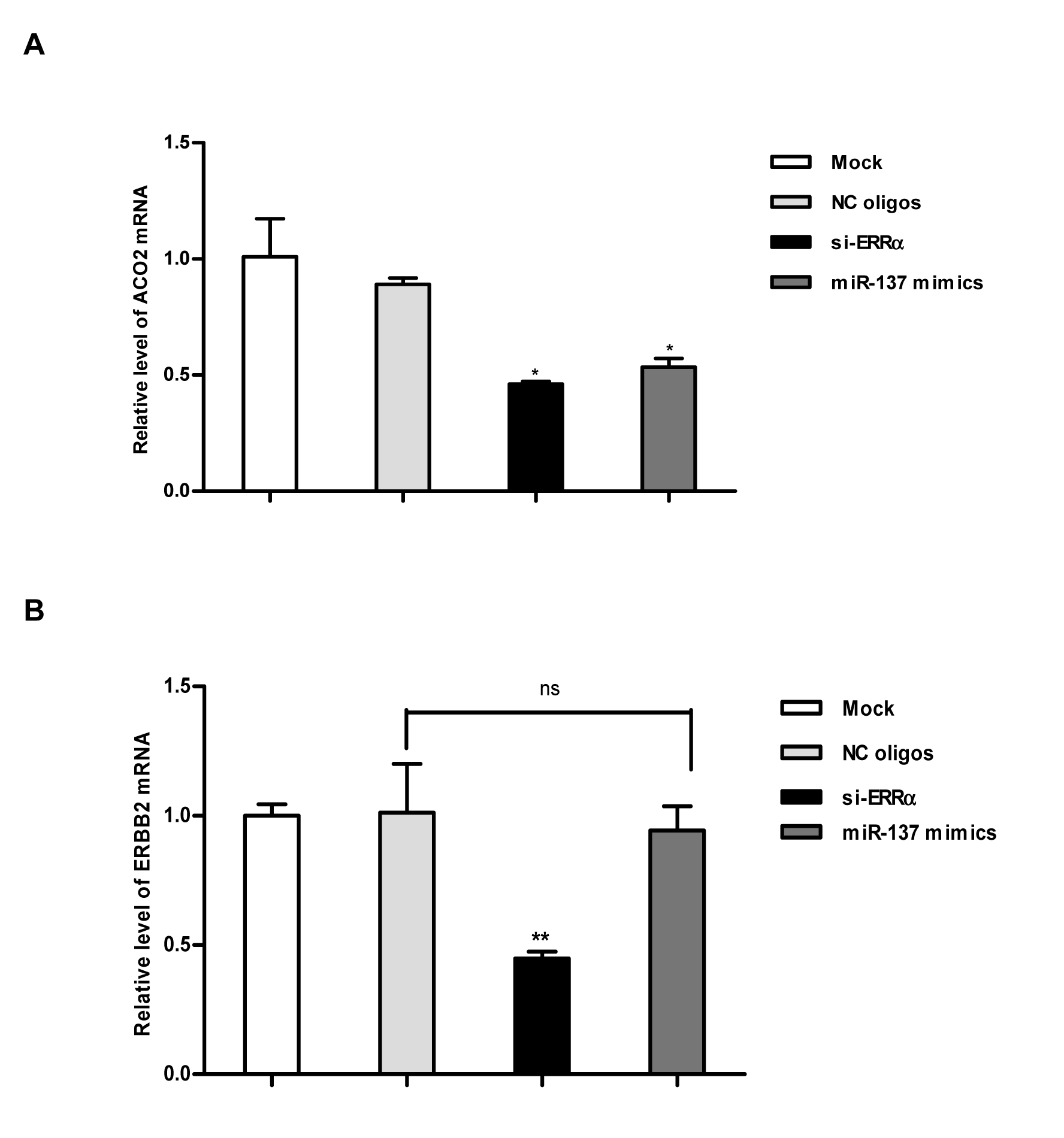

Supplement: Figure S3 — The effect of miR-137 treatment on ACO2 and ERBB2 expression. A. qRT-PCR analysis for ACO2 level in SK-BR-3 cells 48 hr after transfection with NC-oligos, si-ERRα or miR-137 mimics. B. qRT-PCR analysis for ERBB2 level in SK-BR-3 cells 48 hr after transfection with NC-oligos, si-ERRα or miR-137 mimics. ACO2 and ERBB2 expression was normalized to β-actin mRNA expression. The relative expression level of was determined using the 2-△△CT method. Data are representative of three independent experiments performed in triplicate. Error bars: SD; *: p<0.05; **: p<0.01. (TIF) [file pone.0039102.s003.tif]
